# IGF-IR Signal Transduction Protein Content and Its Activation by IGF-I in Human Placentas: Relationship with Gestational Age and Birth Weight

**DOI:** 10.1371/journal.pone.0102252

**Published:** 2014-07-22

**Authors:** Germán Iñiguez, Juan José Castro, Mirna Garcia, Elena Kakarieka, M. Cecilia Johnson, Fernando Cassorla, Verónica Mericq

**Affiliations:** 1 Institute of Maternal and Child Research, University of Chile, Santiago, Chile; 2 Hospital Clínico San Borja-Arriarán, University of Chile, Santiago, Chile; Medical Faculty, Otto-von-Guericke University Magdeburg, Medical Faculty, Germany

## Abstract

**Introduction:**

The human placenta expresses the IGF-I and IGF-IR proteins and their intracellular signal components (IRS-1, AKT and mTOR). The aim of this study was to assess the IGF-IR content and activation of downstream signaling molecules in placentas from newborns who were classified by gestational age and birth weight. We studied placentas from 25 term appropriate (T-AGA), 26 term small (T-SGA), 22 preterm AGA (PT-AGA), and 20 preterm SGA (PT-SGA) newborns. The total and phosphorylated IGF-IR, IRS-1, AKT, and mTOR contents were determined by Western Blot and normalized by actin or with their respective total content. The effect of IGF-I was determined by stimulating placental explants with recombinant IGF-I 10^-8^ mol/L for 15, 30, and 60 minutes.

**Results:**

The IGF-IR content was higher in T-SGA compared to T-AGA placentas, and the IRS-1 content was higher in PT-placentas compared with their respective T-placentas. The effect of IGF-I on the phosphorylated forms of IGF-IR was increased in T-SGA (150%) and PT-SGA (300%) compared with their respective AGA placentas. In addition, AKT serine phosphorylation was higher in PT-SGA compared to PT-AGA and T-SGA placentas (90% and 390% respectively).

**Conclusion:**

The higher protein content and response to IGF-I of IGF-IR, IRS-1, and AKT observed in SGA placentas may represent a compensatory mechanism in response to fetal growth restriction.

## Introduction

Fetal growth is under the control of genetic, environmental, and nutritional factors. Intrauterine growth restriction (IUGR) is an important obstetrical problem and refers to a fetus that has not reached its growth potential [1]. This condition may be the consequence of maternal, fetal, or placental factors. Growth-restricted fetuses/newborns are characterized by increased fetal and neonatal mortality and morbidity [2], [3], as well as preterm birth and risk of chronic disorders in adult life [4], [5].

Recent advances in neonatal care have led to an improvement in the clinical outcome of premature infants (gestational age <37 weeks). Unfortunately, some of these infants develop both [6] early and late morbidities, which may include motor, cognitive, visual, hearing, social-emotional, growth and metabolic problems [7].

The insulin-like growth factors (IGFs) have potent mitogenic activity and appear to be major determinants of fetal growth [8], [9], [10]. These factors are expressed both in the fetus and placenta in most species[11], [12], [13].

IGF-I initiates its biological effects by binding to its cell surface receptor, i.e., IGF-IR [14]. This tyrosine kinase receptor is composed of two heterodimers, which consist of an α- and a β-subunit. Ligand binding to IGF-IR leads the endogenous tyrosine kinase activation resulting in the autophosphorylation of tyrosine residues located in the cytoplasmic regions of the receptor β-subunit, followed by phosphorylation of downstream signaling pathways. One of the most important families of proteins which are phosphorylated by activated IGF-IR are the insulin receptor substrate (IRS) proteins [15], [16]. The activated IRS proteins serve as docking proteins for several signaling molecules, which become activated upon binding. This ultimately results in the activation of at least two main signaling pathways: the Ras/Raf/mitogen-activated protein kinase (MAPK) pathway and the phosphoinositide-3 kinase (PI3K)/AKT/mTOR/p70S6K pathway [17]. Upon activation, these downstream molecules mediate a wide variety of intracellular signals in many cells and tissues, including those regulating glucose transport, protein synthesis, cell proliferation, and survival [17].

The aim of this study was to assess whether IGF-IR and downstream signaling molecules content and activation induced by IGF-I have differences in placentas of different gestational ages and according to birth weight. We also analyzed the associations between the placental protein content and IGF-I induction with birth length and placental weight.

## Materials and Methods

### Sample collection

The placental tissue was collected immediately after delivery. We selected placentas from full term (T: 37–40 weeks of gestation) and preterm newborns (PT: 32–36 weeks of gestation). The newborns were delivered by cesarean section in approximately one third of the cases and their Apgar scores were normal. The newborns with a birth weight between the 10^th^ and the 90^th^ percentiles for gestational age were defined as appropriate for gestational age (AGA), and the newborns with a birth weight below the 10^th^ percentile as small for gestational age (SGA) using Chilean birth weight references [18]. Exclusion criteria were maternal hypertension, diabetes, or a reduced amount of amniotic fluid at delivery. We studied 93 gestations; 25 T-AGA placentas, 26 T-SGA placentas, 22 PT-AGA placentas and 20 PT-SGA placentas. The clinical characteristics of the T-AGA, T-SGA, PT-AGA and PT-SGA neonates are shown in Table 1. All mothers gave their written informed consent and this protocol was approved by the Institutional Review Boards of the San Borja Arriarán Clinical Hospital and the School of Medicine of the University of Chile in Santiago, Chile.

**Table 1:** Anthropometric data for T-SGA, T-AGA, PT-SGA and PT-AGA newborns.

	T-SGA (26)	T-AGA (25)	PT-SGA (20)	PT-AGA (22)
Gestational age (weeks)	38.3±0.2	39.4±0.2	34.1±0.6	34.9±0.3
Gender: males/females	10/16	11/14	11/9	13/9
Birth weight (g)	2621±28*	3418±75	1755±124*	2449±75
Birth weight (SDS)	−1.66±0.07*	−0.07±0.16	−2.12±0.19*	−0.46±0.13
Birth length (cm)	47.2±0.3*	50.2±0.3	40.1±1.2*	45.0±0.8
Birth Length (SDS)	−1.66±0.15*	−0.17±0.17	−2.83±0.51*	−0.79±0.36
Placental Weight (SDS)	531±19*	654±23	405±47*	596±34

Data are expressed as mean ± SEM. * A p value of less than 0.05 was considered statistically significant.

Each placenta was inspected by a pathologist (EK) for any possible abnormalities. Placental villous tissue was collected from preterm and term pregnancies, 30–50 g villous tissue was dissected and quickly washed thoroughly in cold sterile saline solution (NaCl 0.154 mol/L). To study total protein content, placental tissue was dissected free of chorion and decidua into 80–100 mg pieces, washed in sterile saline solution and immediately frozen in liquid nitrogen and stored at −80°C.

### Placental explant cultures

Small fragments of placental tissue (10–20 mg) were dissected from the placenta and washed in ice-cold sterile saline solution. Three fragments per well were placed and cultured at 37°C in 12-well plates for 1 hour in 2.0 ml of DMEM/F-12 (Invitrogen, Life Technologies; Carlsbad, CA, USA) medium containing 100 U/ml penicillin100 µg/ml streptomycin and 0.25 µg/ml amphotericin (Invitrogen, Life Technologies). Subsequently, the medium was changed by fresh DMEM/F-12 medium and the explants were stimulated with 10^−8^ mol/L IGF-I (Austral Biologicals, San Ramon, CA, USA) during 0 (basal), 15, 30 or 60 minutes; this dose of IGF-I was previously determined in our laboratory by testing IGF-I concentrations ranging from 10^−9^ to 10^−6^ mol/L in human placental explants; we selected 10^−8^ M because at this concentration we observed a significant increase in Tyr-IGF-IR (data not shown); in addition this concentration has been employed in previous studies [19]. At each time point the explants were removed, frozen in liquid nitrogen and stored at −80°C.

### Protein extraction

Frozen placental tissue was powdered in a ceramic mortar with liquid N_2_ and homogenized for 30 seconds with a mechanical homogenizer (Kontes Glass Company, Vineland, NJ, USA) in ice-cold Tissue Extraction Reagent 1 (Biosource International, Inc, Camarillo, CA, USA) supplemented with 1% Triton X-100 (Sigma-Aldrich, St Louis, MO, USA) and anti-proteases [Complete, Mini, EDTA-free Protease Inhibitor Cocktail Tablets, Roche Applied Science, Basel, Switzerland)].

The tissue homogenate was incubated for 30 minutes at 4°C with gentle stirring and centrifuged at 10,000 x g for 30 minutes. The resulting supernatant was collected and assayed for protein concentration using the BCA protein assay kit (Pierce, Rockford, IL, USA) with bovine serum albumin (BSA) as standard.

### Western blot analysis

Equal amounts (25 µg) of placental proteins were resolved by electrophoresis using 8% SDS-polyacrylamide gels and then transferred to nitrocellulose membranes (BioRad Laboratories, Hercules, CA). The membranes were blocked with 5% BSA in TBS-T (20 mmol/L Tris pH 7.2, 137 mmol/L, NaCl, 0.1% (v/v) and Tween-20) for 1 h at room temperature. Blots were probed with antibodies against total IGF-IRβ, IRS-I and AKT [Santa Cruz Biotechnology, Santa Cruz, CA, USA]; mTOR (Cell Signaling, Danvers, MA, USA), phospho-IGF-IR-Tyr1161, (Abcam,Cambridge, England), phospho-IRS-1- tyr1229, AKT-Ser473 and AKT-Thr308 (Santa Cruz Biotechnology,) and phospho-mTOR-Ser2481 (Cell Signaling). Anti β-actin (Sigma-Aldrich) was used to normalize the different placental protein content. After extensive washing, bands were detected with the appropriate horseradish peroxidase-conjugated secondary antibodies (Rockland Immunochemical Research, Gilbertsville, PA, USA), followed by enhanced chemiluminescence (ECL plus Western Blotting Detection System, Amersham Biosciences, Bucking Hanshire, UK).

The images were acquired and evaluated by scanning densitometry using the UltraQuant Image Acquisition and Analysis Software (Ultralum Incorporated, Claremont, CA, USA); specific times of exposure and settings were established for each protein. Total protein content band intensity was expressed in arbitrary units (optic densitometry units, AU) and normalized relative to β-actin content. The activation induced by IGF-I of each protein was obtained by the ratio of phosphorylated-protein/total protein content at each time point.

### Statistical analysis

Results are shown as mean ± SEM. Differences within each group (T-AGA, T-SGA, PT-AGA and PT-SGA) were assessed by one-way ANOVA or Kruskall-Wallis, followed by the Bonferroni test for multiple comparisons. According to the distribution of the data, correlations were established using the Pearson or Spearman test. Statistics were performed using SPSS v21, and a value of p<0.05 was considered significant.

## Results

Clinical data of the subjects studied and placental weight are shown in Table 1. As expected, birth weight, birth length and placental weight from SGA newborns was significantly lower than their AGA counterparts.

### *Ex vivo* Placental Total protein content

**Figure 1:**
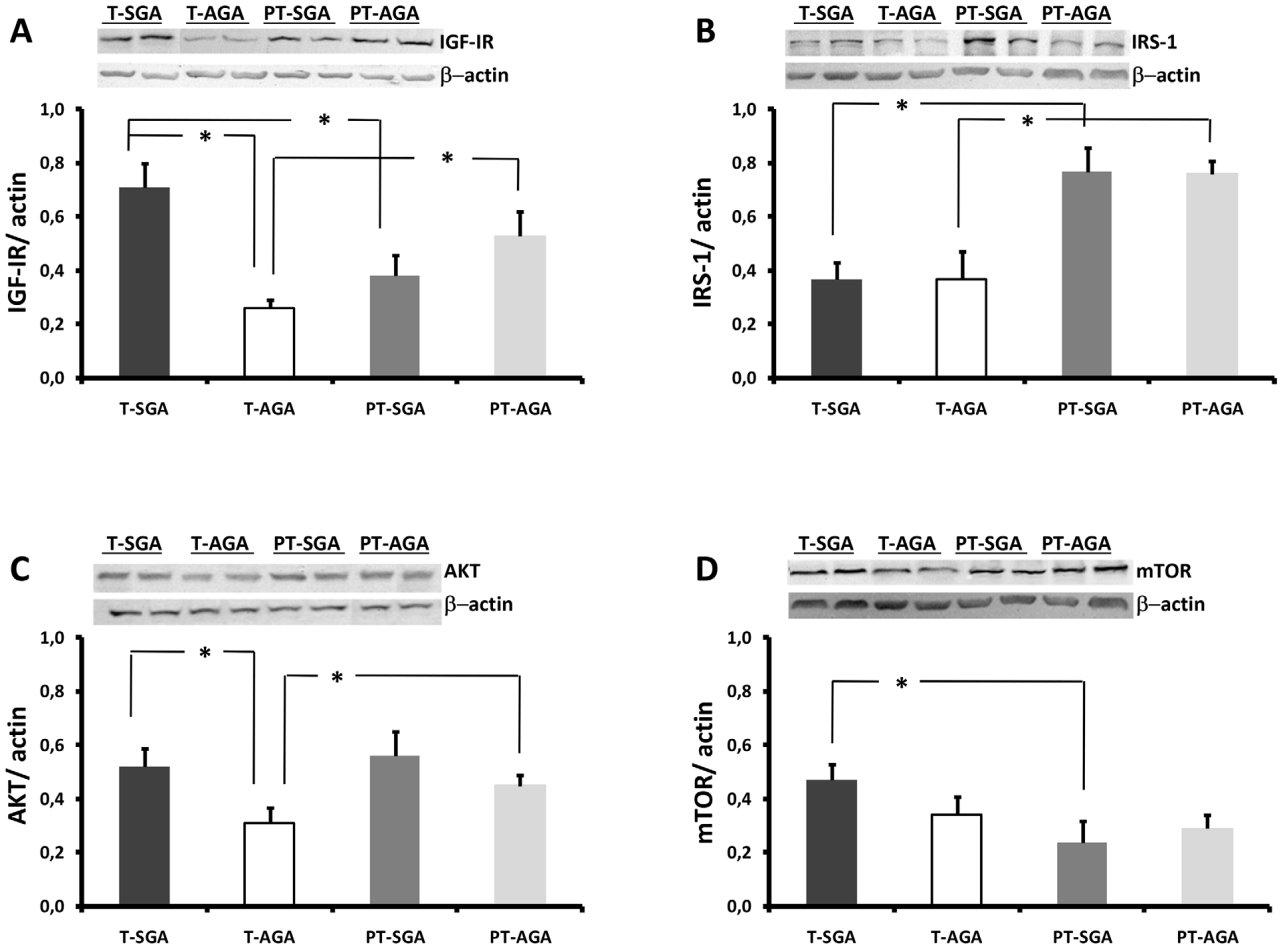
Placental IGF-IR (A), IRS-1 (B), AKT (C), and mTOR (D) obtained from T-SGA (n = 26), T-AGA (n = 25), PT-SGA (n = 20) and PT-AGA (n = 22) pregnancies. Representative electrophoretic gel for each protein is included in each graph. A p value of less than 0.05 was considered statistically significant.

The total protein contents of IGF-IR, IRS, AKT and mTOR from SGA and AGA placentas is shown in Figure 1. The protein content of IGF-IR was higher in T-SGA placentas compared with T-AGA (170%; p<0.001) and PT-SGA placentas (82%; p = 0.014); we also observed a higher IGF-IR content in PT-AGA compared with T-AGA placentas (103%; p = 0.027) (Figure 1A).

The content of IRS-1 content was higher in PT-SGA compared with T-SGA placentas (110%; p<0.001) and in PT-AGA compared with T-AGA placentas (105%; p<0.001) (Figure 1B).

The AKT placental content was higher in T-SGA compared to T-AGA placentas (67%; p = 0.047), but not between preterm placentas. We also observed a higher AKT content in the PT-AGA compared to T-AGA placentas (45%; p = 0.012) (Figure 1C).

The mTOR content was similar in SGA and AGA placentas from term and preterm newborns. However, we found higher mTOR protein content in T-SGA compared with PT-SGA (105%; p = 0.008) (Figure 1D).

### Effect of IGF-I on IGF-IR, AKT and mTOR activation

**Figure 2:**
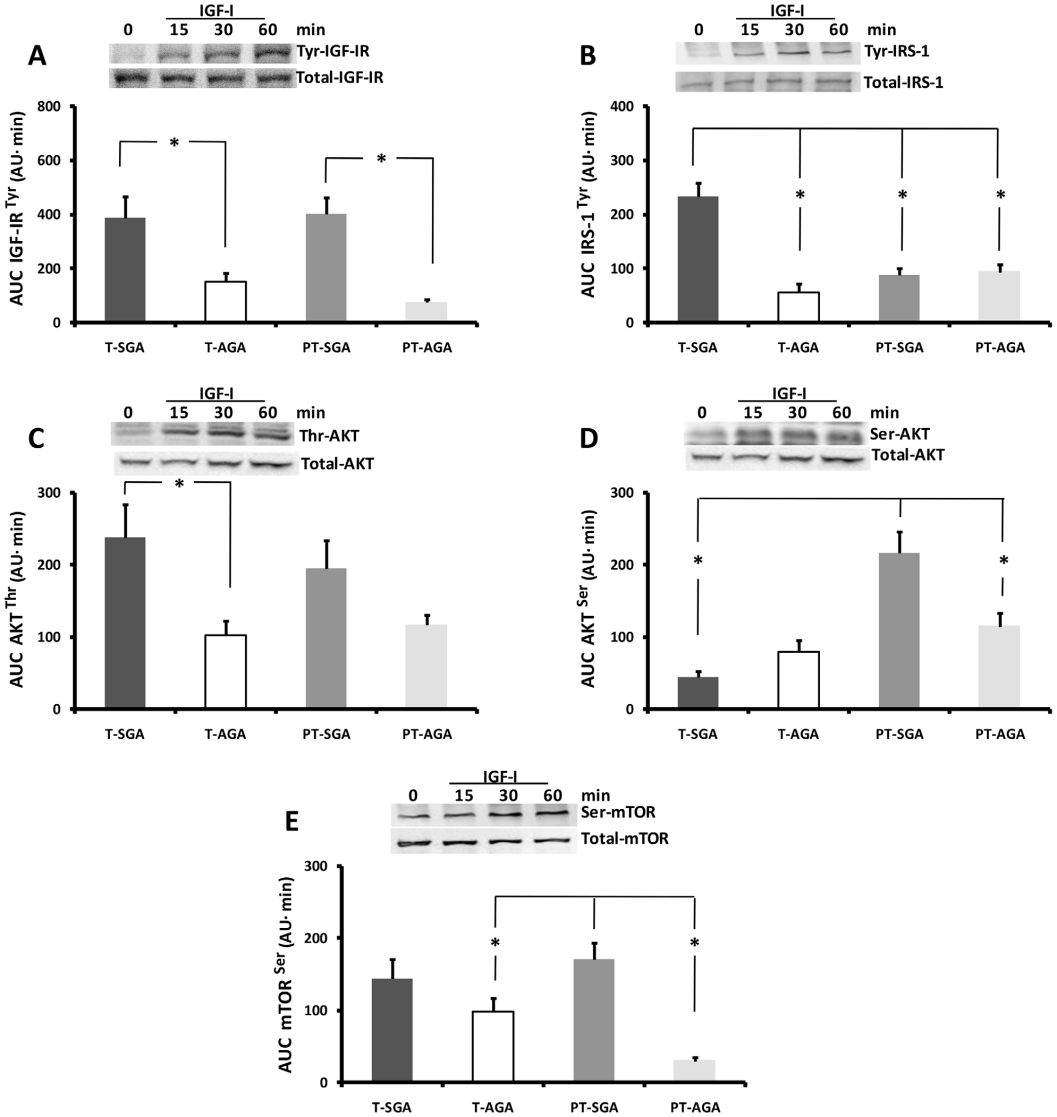
Activation of Tyr-IGF-IR (A), Tyr-IRS-1(B), Thr-AKT (C), Ser-AKT (D) and Ser-mTOR (E) with IGF-I 10-8 mol/L in placental explants from T-SGA, T- AGA, PT-SGA and PT-AGA newborns. Representative electrophoretic gel for each protein is included in each graph. The activation is expressed as area under the curve AUC. A p value of less than 0.05 was considered statistically significant.

We studied the effect of stimulation with IGF-I 10^−8^ mol/L for 60 min on the phosphorylation of IGF-IR, AKT, and mTOR in explants from term and preterm placentas. The integrated activation of each protein is shown in the Figure 2 as the area under the curve (AUC), calculated by the trapezoidal rule.

The activation of IGF-IR was higher in T-SGA (155%; p = 0.047) compared with T-AGA and in PT-SGA compared with PT-AGA (300%; p<0.001) placentas (Figure 2A). The tyrosine IRS-1 activation induced by IGF-I was higher in T-SGA compared with T-AGA placentas (314%; p<0.001) and compared with PT-SGA (165%; p<0.001) placentas, but it was lower in T-AGA when compared with PT-AGA placentas (68%; p = 0.013) (Figure 2B).

AUC phosphorylation of placental threonine AKT after one hour of incubation with IGF-I was higher in placentas from SGA compared to AGA newborns (131%; p = 0.033) (Figure 2C). The AUC for serine-AKT was higher in PT-SGA compared with PT-AGA (90%; p = 0.012) and with T-SGA placentas (390%; p<0.001) Figure 2D).

There were no differences in the activation of placental mTOR induced by IGF-I (Figure 2E) in the placentas from term newborns, but it was higher in PT-SGA compared with PT-AGA placentas (470%; p<0.001), and in T-AGA compared to PT-AGA placentas (230%; p = 0.001).

### Correlation of placental protein contents and IGF-I responses with birth weight, birth length and placental weight

The correlations between IGF-IR protein content and signaling molecules with birth weight, birth length and placental weight are shown in Table 2, and the correlations between the activation of these proteins after stimulation with IGF-I with birth weight, birth length and placental weight are shown in Table 3. We observed an inverse correlation between the content and activation of IGF-IR, IR and AKT with birth weight and birth length (SDS).

**Table 2:** Correlations between: birth weight, birth length and placental weight with placental protein content.

	Birth Weight (SDS)	Birth Length (SDS)	Placental Weight (g)
IGF-IR	−0.272*	−0.214*	−0.161
IRS-1	−0.056	−0.127	−0.126
AKT	−0.284*	−0.335*	−0.247*
mTOR	−0.054	0.015	0.031

* A p value of less than 0.05 was considered statistically significant.

**Table 3:** Correlations between birth weight, birth length and placental weight with activated proteins in placental explants.

	Birth Weight (SDS)	Birth Length (SDS)	Placental Weight (g)
AUC Tyr-IGF-IR	−0.239*	−0.250*	−0.106
AUC Tyr-IRS-1	−0.420*	−0.380*	−0.243
AUC Thr-AKT	−0.377*	−0.277*	−0.177
AUC Ser-AKT	−0.244*	−0.179	−0.068
AUC Ser-mTOR	−0.442*	−0.235	−0.196

* A p value of less than 0.05 was considered statistically significant. [AUC]  =  area under curve).

## Discussion

To our knowledge this is the first study that investigates the IGF-IR signal transduction pathway in human preterm and term placentas from SGA and AGA newborns. In addition, we studied the activation of these placental proteins induced by IGF-I. We observed differences in the protein content and activation of the IGF-IR signal transduction pathway according to gestational age and birth weight.

The increased IGF-IR content observed in T-SGA compared with T-AGA placentas has been previously described by our group [20]. However, this difference was not found in the preterm group, perhaps due to a maturational compensatory process to enhance growth that it is not ongoing at that gestational age. The higher IGF-IR protein content observed in SGA placentas is in concordance with some studies but not with others [21], [22], [23]. These differences are probably related to the different etiologies of the SGA newborns studied in each series, but in particular, by their length of gestation as suggested by our results. In one of these studies, they compared preterm SGA with term AGA placentas [22] and in another study [23] the authors analyzed placentas from comparable gestational ages of approximately 36 weeks. The *in vitro* IGF-IR activation induced by IGF-I showed a similar behavior, with both T-SGA and PT-SGA placentas showing a higher activation compared with their respective AGA placentas. These findings suggest that the higher receptor content and activation induced by IGF-I represent a possible compensatory mechanism of the placenta in response to fetal growth restriction in both term and preterm gestations.

In addition, our study showed a higher IRS-1 protein content in placentas from premature newborns. However, we observed a higher tyrosine activation of IRS-1 in response to IGF-I in T-SGA compared with the other groups of placentas, Two studies have described a higher basal (*ex vivo*) IRS-1 phospho protein in AGA compared to SGA placentas [22], [23]. The increased basal phosphorylation of IRS reported by these authors in placenta, does not necessarily represent the responsiveness of the placental tissue to stimulation with IGF-I. It is interesting to consider the significant differences in IRS-1 protein content observed in the placentas from preterm compared with term pregnancies. We also observed an increased activation of tyrosine-IRS-1 in SGA placentas, particularly from term newborns, suggesting that following acute IGF-I stimulation, IRS-1 is phosphorylated on tyrosine residues to propagate IGF-I signaling, as has been observed in other experimental models [24], [25], [26].

The AKT activation by IGF-I is a multistep process involving translocation and phosphorylation. Two phosphorylation sites, Thr308 and Ser473, appear to be critical for the activation of AKT induced by growth factors [27]. Phosphorylation of Thr308 in the activation loop by PDK1 is essential for AKT activation, and of Ser473 at the C-terminal tail by either autophosphorylation, or by PDK2 for maximal activation of kinase activity [27].

Although total protein placental AKT and Thr-AKT phosphorylation were higher in T-SGA compared to T-AGA placentas, we observed an increased Ser-AKT in PT-SGA, compared to T-SGA and PT-AGA placentas, suggesting another possible compensatory placental mechanism in response to fetal growth restriction. As mentioned, the Thr308 phosphorylation activates partially AKT, but for complete activation, the phosphorylation of Ser473 is required for regulating the function of several cellular proteins involved in glucose [28] and amino acid [29] metabolism, survival/apoptosis, cell differentiation and proliferation [30]. The fully Ser-AKT phosphorylated form induced by IGF-I in PT-SGA placentas, suggests that this placental compensatory mechanism is probably more important in preterm pregnancies.

The mTOR protein is an evolutionally conserved serine/threonine kinase that integrates signals from multiple pathways [31], including nutrients (amino acids and glucose) [32], growth factors [29] (insulin and IGF-I), hormones [33] (e.g., leptin), and different stresses [34] (e.g., starvation, hypoxia, and DNA damage). It regulates a wide variety of eukaryotic cellular functions, such as transcription, translation, transcription, protein turnover, cell growth, differentiation, metabolism, energy balance, and stress response [35]. This suggests that mTOR is involved in the uptake of amino acids during pregnancy for fetal development. We did not find differences in placental mTOR contents between T-SGA and PT-SGA compared with their respective AGA placentas, but the mTOR content was higher in T-SGA compared to PT-SGA placentas. The greater activation of mTOR induced by IGF-I in PT-SGA placentas suggests that this molecule is more sensitive to IGF-I in preterm SGA placentas. The fact that no differences were observed in the activation of mTOR in placentas from term newborns indicates that this molecule is a key component of placental IGF-I signaling during early gestation and may regulate fetal growth.

Interestingly, most protein contents and their activation by IGF-I were inversely related with birth weight and birth length, suggesting that this placental signal transduction pathway plays an important role in fetal growth. The inverse relationship between fetal weight with IGF-IR, IRS-1, AKT and mTOR placental content and with the activation of these proteins induced by IGF-I, suggest that this placental signal transduction pathway plays an important role in fetal growth.

In conclusion, we describe for the first time that the IGF-IR/IRS-1/AKT/mTOR protein contents, as well as their activation induced by IGF-I in human placental explants are up-regulated in term and preterm SGA compared to AGA placentas. In addition, we observed an inverse correlation between birth weight and the placental content, as well as the activation of these proteins. These findings may represent a compensatory placental mechanism in response to fetal growth restriction.
